# Toward more mindful reporting of patient and public involvement in healthcare

**DOI:** 10.1186/s40900-021-00308-8

**Published:** 2021-09-09

**Authors:** Brett Scholz, Alan Bevan

**Affiliations:** 1 ANU Medical School, Ngunnawal Country, The Australian National University, 54 Mills Rd, Acton, ACT 2601 Australia; 2 School of Psychology, Kaurna Country, North Terrace, The University of Adelaide, Adelaide, SA 5000 Australia

**Keywords:** Patient partners, Research reporting, Checklists, Consumer leadership, Consumer researchers, Lived experience leadership, Lived experience researchers

## Abstract

Understanding of the value of patient and public involvement in research has grown in recent years, but so too has uncertainty about how best to practice and how best to report such involvement in research outputs. One way proposed to report such involvement is through checklists, such as the GRIPP2, which aims to improve quality, transparency, and consistency in such reporting. We critique the unproblematised use of such a tool because of two main concerns. First, being asked to complete a GRIPP2 for a recent publication felt divisive given that the service user researcher was as much a member of the authorship team as the other researchers (whose involvement did not necessitate a checklist). Second, checklists do not actually address the power imbalances and tokenism that is rife in patient and public involvement in research. Indeed, the false sense of objectivity fostered by meeting the minimum requirements of the checklist means that researchers may not go further to engage in reflexive research practices and reporting. Rather than rote use of such checklists, we recommend mindful reflexive reporting in research outputs of patient and public involvement processes. We also recommend future iterations of the GRIPP consider (a) incorporating criteria about whether the checklist is completed by or with service user researchers or not, (b) addressing criteria that position service user research as needing to be justified, and (c) expanding the “critical perspective” element of the checklist to explicitly consider power differentials.

## Plain English summary

Checklists (such as the GRIPP2) have been proposed as a way to improve how research papers report patient and public involvement in research projects. We were recently asked to complete a GRIPP2 as part of a peer review process, and it felt divisive. AB—a service user researcher—was just as much a part of the research team as was BS—a critical health psychologist. However, the role of BS in the research did not have to be justified or reported in the same way that was being asked of AB. A further concern about being required to use the checklist was that it did not necessarily lead to better reporting. Rather, it created a false sense of objectivity about patient and public involvement, and this might serve to hide power imbalances and tokenism in the research process. We recommend that instead of relying on such checklists, that researchers, reviewers, editors and readers reflect on patient and public involvement processes and the ways in which these are reported. We also make recommendations to make future iterations of the GRIPP more inclusive.

We are glad to see that the importance of patient and public involvement and engagement across all levels of health systems has become better understood in recent years, and that such involvement and engagement is now a policy imperative in many jurisdictions [1]. We note too, though, that rhetoric about patient and public involvement is becoming more common across medical disciplines, countries, and types of research. With this increasing understanding that such involvement is important also comes some uncertainty about best practices about involving service users. Evidence suggests involvement often remains peripheral and rarely includes meaningful collaborations between service user researchers and other researchers as equals [2]. Further, there appears to be uncertainty and potentially misleading *reporting* about involving service users. For instance, we have been concerned about research reports published in which researchers claim to have *involved* service users as “co-researchers” without co-authorship with (or even acknowledgement of) their service user researcher colleagues [3].

Involvement itself is a broad term in research, ranging from practices in which service users have no power (e.g., service user researchers being ‘used’ to endorse other researchers’ decisions) through to co-produced (in which decision-making power and agenda-setting are shared) or patient-led (in which decision-making power rests with patients) research [4]. Further complicating this is the range of terms used in particular contexts and jurisdictions and by different individuals to refer to service user researchers (such as Expert by Experience, Consumer Researcher, or Lived Experience Researcher just for some common examples). Given myriad types and understandings of involvement, it is understandable that there are many ways that such partnerships are reported in research. However, discordance between researchers’ claims about and practices of involvement could be adding to this confusion.

One of the ways proposed to address this issue is through checklists. Arguably one of the more common of these checklists relating to research collaborations with service users is the GRIPP2 [5], which now has over 500 citations on Google Scholar. The GRIPP2 was developed to address poor quality reporting through improving the quality, consistency, and transparency of the evidence base for patient and public involvement in research [5]. Despite this commendable aim, we are concerned that such checklists may provide a false sense of objectivity about such partnerships, do not necessarily improve the evidence base for patient and public involvement research, and may in fact be divisive and position service user led or coproduced research as abnormal, and we outline each of these concerns below.

## False sense of objectivity

An issue related to checklists more broadly is that they can create a false sense of objectivity about research reporting [6]. In the case of the GRIPP2, the checklist provides researchers with a set of statements about reporting of involvement, in turn implying that such involvement was objective by ignoring some of the most important issues in collaborative research: the redressing of power imbalances and the negotiation of roles and practices [4, 7].

We could not find any guidelines from journals or researchers suggesting that service user researchers should be involved in the completion of GRIPP2 forms. We suspect that instead of being conducted by or with service user researchers, in most cases this is often seen as an administrative task that other (non-service user) researchers complete prior to article submission. One way in which research teams could be more reflexive about collaborative practices is to complete GRIPP2 forms as a team, or to compare GRIPP2 responses from service user researchers and other team members. This may help to improve future collaborations, and remove some concern about checklists creating any false sense of objectivity.

## Impact on the evidence base

One of the motivations for the development of the GRIPP2 was to address unsystematic reporting of patient and public involvement which has created problems for systematic reviews that attempt to synthesise these works [5]. However, in a recent systematic review of reviews conducted with service user co-researchers, only 1 of 37 included studies used such a reporting framework [8]. Further, service user researcher involvement was often only indicated in the author affiliation field *or* in the review text itself [8]. For instance, one included study was our systematic review in which AB’s affiliation was given as “Consumer Researcher” [3]. This aligns with other contemporary suggestions such as using the PubMed “Patient Author” affiliation tag to provide a more systematic approach to enable searching for this work [9]. Thus it appears that the GRIPP2 may not be meeting its aim to address unsystematic reporting of patient and public involvement.

Relatedly, another of the underlying issues that precipitated the development of the GRIPP forms is still a problem today: there is a lack of MeSH (Medical Subject Headings) to support the better indexing and searching of research conducted with service user researchers [10]. There are MeSH terms related to ‘patient participation’ but these do not currently differentiate between research *about* participation in health, and participatory research. Given the growing understanding of the importance of producing research together with service users, we recommend revisions to MeSH terms to improve the way such work is indexed.

## Positioning

Recently, an anonymous peer reviewer asked us to consider completing a GRIPP2 to document AB’s role in a jointly written manuscript [3]. The request felt divisive given that AB as a service user researcher was as much a member of the authorship team as the psychologist (BS), the biostatistician, the palliative care nurse, and the ICU physician—and we are never asked in multidisciplinary health research to justify or explain the contribution of, for instance, our nursing colleagues to research papers. Thus being required to complete a GRIPP2 may instead position service user-led or coproduced research as abnormal.

Some of the GRIPP criteria may be particularly problematic here. For instance, the criterion that asks researchers to “report the aim of public and patient involvement in the study” is one that seems to specifically position such collaborative research as needing justification. It does not seem appropriate to ask this of work that is genuinely led by or co-produced with service user researchers—just as other researchers are not asked to justify their role as a researcher in any given study.

## Ensuring the GRIPP2 does not replace reflexive reporting

Redressing power imbalances and tokenism in collaborations with service user researchers is vital [11], but the GRIPP2 does little to address these issues—and the way it positions collaboration with service user researchers as unusual (compared to collaborations with other multidisciplinary colleagues) may amplify these issues. Indeed, the checklist does not require authors provide reflection on power relations—one of the key aspects of such collaboration [4]. The GRIPP2 itself purports to have been developed in part through the involvement of patients and the public (“patients were involved as research partners in all aspects of the study”). However, the extent to which the GRIPP2 development was based on partnership with service users in epistemic decision-making and agenda-setting roles is not made explicit [5], highlighting how use of the checklist does not address these fundamental concerns about the role of power in collaborations with service users.

Although it is more than two decades since the first dedicated “consumer academic” role was established [12], and more than 15 years since the concept of patient leaders was introduced in academia [13], collaboration between service users and other researchers is still often considered novel. As such, the motive for developing the GRIPP2 should be commended—collecting evidence about effective collaborations might indeed promote better partnerships. Indeed, the GRIPP2 could act as a pointer to encourage researchers to begin to see and address their limitations in engaging people with lived experience more meaningfully in research.

The flexibility of the GRIPP2 means that researchers may be asked by editors or reviewers to complete the checklist for a study anywhere on the spectrum of involvement—from service user led research on one end, to a project in which only consultation with consumers was attempted at the other. Thus, we recommend that the use and interpretation of the checklist be done reflexively. We suggest that researchers, editors, reviewers, and readers consider carefully the philosophy underpinning collaborative research and remember that simply using the GRIPP2 does not necessarily result in good involvement practices being followed. We hope that authors—whether they choose to report using the GRIPP2 or not—reflexively report in their publications about how patient and public involvement influenced the research—including, where appropriate, how challenges were overcome and how power relations were acknowledged and addressed—as these would be beneficial learnings for any researcher wanting to improve their practices in patient and public involvement. Without being prescriptive of any one particular way in which this should be reported, there is one example that stands out of what this might look like. In the cited paper, the subsection entitled “Research Team” [14] discusses how—based on the principles of co-production—power imbalances in the study were addressed through equal numbers of consumer researchers and other researchers.

Given the broad spectrum of patient and public involvement in health care, a one-size-fits-all approach such as the GRIPP2 can allow tokenism to remain hidden, can reproduce the idea that involvement of patients and the public is *unusual*, and can privilege the clinical or theoretical knowledge of other researchers over the experiential expertise of service user researchers. To begin to address these concerns, we recommend that future iterations of the GRIPP consider

- Asking respondents whether the form was completed by or with service user researchers,
- Exploring further ways in which some criteria of the GRIPP may be more divisive—for instance asking researchers to “report the aim of public and patient involvement in the study” is one such criterion which implies that some kind of justification needs to be provided, and
- Expanding on the “critical perspective” criterion of the checklist to explicitly ask authors to consider and report ways in which power differentials were acknowledged, explored, and addressed.

We strongly advocate for more mindful use and consumption of the GRIPP2 by authors, editors, reviewers, and readers. We also encourage authors to discuss openly and in detail whether they met the goals of patient and public involvement, and to strive beyond involvement for co-produced and service user-led research.

## Data Availability

Not applicable.
